# Risk of multiple primary cancers in patients with atypical fibroxanthoma/pleomorphic dermal sarcoma: A SEER analysis

**DOI:** 10.1016/j.jdin.2024.08.003

**Published:** 2024-09-02

**Authors:** Vraj K. Shah, Rajiv I. Nijhawan

**Affiliations:** a University of Texas at Southwestern Medical School, Dallas, Texas; b Department of Dermatology, University of Texas Southwestern Medical Center, Dallas, Texas

**Keywords:** clinical research, epidemiology, general dermatology, medical dermatology, oncology, surgical dermatology

*To the Editor:* Atypical fibroxanthoma (AFX) and pleomorphic dermal sarcoma (PDS) are malignant mesenchymal dermal neoplasms considered to be on a spectrum of disease severity.^1^ While both AFX and PDS are locally aggressive, AFX rarely metastasizes, while PDS is associated with a high risk of recurrence and metastasis.^1^^,^^2^ AFX/PDS classically presents in elderly patients on sun-exposed or previously radiated surfaces as a solitary, red nodule or papule.^3^^,^^4^ AFX has a 5-year recurrence rate around 7%,^2^ and the risk of transformation from AFX to PDS after local AFX recurrence is 13%.^2^ Given the mesenchymal nature of AFX/PDS, we sought to determine whether AFX/PDS contributes to an increased risk for secondary malignancies.

This cohort study utilized the Surveillance, Epidemiology, and End Results (SEER) database from the National Institutes of Health. This data contained 17 de-identified regional registries across the United States (excluding Alaska) from January 1, 2000, to December 31, 2020. Patients were included based on a primary diagnosis of AFX or PDS limited to a cutaneous site. In total, 2140 patients were identified. The risk of secondary malignancies following diagnosis were quantified using the standardized incidence ratio (SIR) (Ratio of observed to expected cases for subsequent cancers.) and the excess risk per 10,000 person-years (Difference between observed and expected cases of subsequent cancers divided by the person-years at risk.).

The average age at primary diagnosis of AFX or PDS was 75.3 years. Overall, malignancies at all sites had an SIR of 1.48 (*P* < .05) (Table I). Cutaneous malignancies excluding basal cell carcinoma and squamous cell carcinoma had a significantly increased SIR (*P* < .05). Of these, melanoma of the skin had an increased SIR of 3.43, and other nonepithelial skin malignancies (The classification other nonepithelial skin malignancies include 85 skin cancers diagnoses.) had an SIR of 13.22 (*P* < .05) (SEER does not track subsequent cutaneous basal cell and squamous cell carcinomas likely due to high incidence.). All solid tumors had a significantly increased SIR of 1.38 (*P* < .05). Eye and orbit nonmelanoma malignancies had the greatest SIR following AFX/PDS (SIR – 14.22; *P* < .05). In addition, thymus, adrenal gland, and other endocrine malignancies had a significantly increased SIR (SIR–9.13, *P* < .05). Other significantly increased SIRs included soft tissue malignancies including the heart, esophagus, sigmoid colon, extra nodal non-Hodgkin’s lymphoma, colon excluding rectum, and miscellaneous (*P* < .05). The increased risk of nonmesenchymal malignancies highlights the potential role of impaired DNA damage repair in AFX/PDS development.^5^ Cutaneous malignancies represented the greatest excess risk including excess risk of melanoma of the skin (60.41, 36.79; respectively). Soft tissue malignancies including the heart, followed by miscellaneous malignancies represented the greatest excess risk for solid tumor malignancies (excess risk: 11.60 and 11.02, respectively). Among females, there was also a significant increase in respiratory system, pancreas, and lymphatic/hematologic malignancies. AFX/PDS was not significantly protective of any malignancies.

**Table I:** Selected primary cancers with significantly increased risk following AFX/PDS diagnosis from 2000 to 2020

Selected primary cancers	Number of subsequent cancers		SIR∗	95% CI	Excess risk per 10,000 person-years
	Observed	Expected			
All sites	424	286.84	1.48	1.34-1.63	113.03
All sites excluding nonmelanoma skin	393	284.5	1.38	1.25-1.52	89.41
All solid tumors	330	239.67	1.38	1.23-1.53	74.44
Skin excluding basal and squamous cell carcinoma	94	20.7	4.54	3.67-5.56	60.41
Melanoma of the skin	63	18.35	3.43	2.64-4.39	36.79
Other nonepithelial skin	31	2.35	13.22	8.98-18.76	23.61
Colon and rectum	38	26.54	1.43	1.01-1.97	9.45
Colon excluding rectum	31	20	1.55	1.05-2.20	9.06
Miscellaneous	29	15.63	1.86	1.24-2.66	11.02
Soft tissue including heart	16	1.92	8.32	4.75-13.50	11.6
Esophagus	11	3.98	2.76	1.38-4.94	5.78
Sigmoid colon	10	4.36	2.3	1.10-4.22	4.65
Non-Hodgkin’s lymphoma—extra nodal	10	4.54	2.2	1.06-4.05	4.5
Eye and orbit	3	0.48	6.24	1.29-18.24	2.08
Eye and orbit—nonmelanoma	2	0.14	14.22	1.72-51.38	1.53
Thymus, adrenal gland, and other endocrine	2	0.22	9.13	1.11-32.99	1.47

*AFX*, Atypical fibroxanthoma; *PDS*, pleomorphic dermal sarcoma; *SIR*, standardized incidence ratio.

∗ (*P* < .05).

Given that the primary diagnosis of AFX/PDS represents a significantly higher risk of secondary malignancies, further analysis is warranted to identify trends in timeline for secondary malignancy. Clinicians should use these findings to develop appropriate follow-up and referral protocols.

## Conflicts of interest

None disclosed.
